# Exploring trends in benzodiazepine-positive fatal drug overdoses in Tennessee, 2019–2021

**DOI:** 10.1080/07853890.2023.2287194

**Published:** 2023-12-01

**Authors:** Jenna Moses, Jessica Korona-Bailey, Sutapa Mukhopadhyay

**Affiliations:** a CDC Foundation, Atlanta, GA, USA; b TN Department of Health, Office of Informatics and Analytics, Nashville, TN, USA

**Keywords:** Fatal drug overdose, benzodiazepines, state unintentional drug overdose reporting system, Tennessee, surveillance

## Abstract

**Background:**

Benzodiazepine-positive overdoses increased between 2019 and 2021 in Tennessee. We sought to determine the changes in the number and characteristics of prescription and illicit benzodiazepine-positive fatal drug overdoses during this period.

**Materials and Methods:**

A statewide study was conducted to determine changes in the number and characteristics of benzodiazepine-positive drug overdose decedents using 2019–2021 data from the Tennessee State Unintentional Drug Overdose Reporting System. The analyses were limited to Tennessee residents aged ≥ 18 years. A benzodiazepine-positive overdose was defined as any benzodiazepine on toxicology, regardless of the presence of other substances. Frequencies were generated to compare demographics, circumstances, prescription history, and toxicology between 2019 and 2021 for illicit and prescription benzodiazepine-positive fatal overdoses.

**Results:**

Between 2019 and 2021, 1666 benzodiazepine-positive unintentional or undetermined fatal drug overdoses out of 5916 total overdoses that occurred among adult Tennessee residents with available toxicological information. Prescription benzodiazepines were identified in 80.7% of deaths, whereas illicit benzodiazepines were identified in 12.0% of deaths. Many decedents had an anxiety disorder (45.5%), while over half of all decedents had a history of substance use disorder (52.3%). Most benzodiazepine-positive overdoses involved fentanyl (71.3%).

**Conclusions:**

This analysis can inform local and regional public health workers to implement focused prevention and intervention efforts for people with co-occurring mental health conditions and substance use disorders to curb overdose epidemics among persons using benzodiazepines in Tennessee. Public health campaigns should focus on educating people on appropriate prescription medication use and the dangers of obtaining substances illicitly. Given the high proportion of opioids in this population, further education also is needed on the dangers of polysubstance drug use. The differences between prescription and illicit benzodiazepine-positive fatal overdoses indicate the need to develop substance-specific prevention and treatment strategies.

## Introduction

Since 2013, the United States (U.S.) has experienced an exponential increase in morbidity and mortality due to synthetic opioids, particularly fentanyl [1]. Despite the dominance of synthetic opioids, most overdose deaths involve more than one substance. Benzodiazepines are frequently identified in polysubstance overdose deaths, and are often prescribed for anxiety, insomnia, and seizures [2,3]. Common prescription benzodiazepines include alprazolam (Xanax), diazepam (Valium), and lorazepam (Ativan) [4]. Growing concern over polysubstance overdose is partially driven by the emergence of novel psychoactive substances (NPS), including illicit benzodiazepines [5,6]. Illicit benzodiazepines have increasingly been identified in both nonfatal and fatal overdoses over the last 10 years [7]. Data from the National Poison Data System found that there was a 330% increase in illicit benzodiazepine exposure from 2014 to 2017 [8]. Between 2019 and 2020, fatal overdoses involving illicit benzodiazepines increased 519.6%, compared to 21.8% for prescription benzodiazepines [9].

The United Nations Office on Drugs and Crime define NPS as “substances of abuse, either in a pure form or a preparation, that are not controlled by the 1961 Single Convention on Narcotic Drugs or the 1971 Convention on Psychotropic Substances, but which may pose a public health threat. [10]. NPS may be analogs of existing licit substances or newly synthesized chemicals that mirror the effects of controlled substances [11]. NPS benzodiazepines, also known as illicit benzodiazepines, were first reported in the U.S. in 2005 with the identification of phenazepam by the DEA’s National Forensic Laboratory Information System (NFLIS) [12]. Illicit benzodiazepines are easier to access than prescription benzodiazepines since they do not require a prescription and can be easily purchased through the online marketplace [13]. This aligns with reports from people who use NPS who cite ease of access, availability, low cost, fewer risks, and lack of detection by drug tests as primary reasons for NPS use over traditional drugs [14]. Illicit benzodiazepines may also be used as adulterants in the illicit drug supply because they are cheap and readily available, which may achieve similar effects for consumers at a lower cost to suppliers [15]. The recent increase in illicit benzodiazepines on the drug market may have been prompted by a shortage in traditional drugs of abuse during the COVID-19 pandemic [16].

Despite the increase in illicit benzodiazepines, prescription benzodiazepines still account for most benzodiazepine-positive fatal drug overdoses. [9] The percentage of U.S. adults filling a benzodiazepine prescription increased by about 30% between 1996 and 2013, corresponding to a four-fold increase in the rate of benzodiazepine-positive fatal overdoses between 1996 and 2010 [17]. Although fatal overdoses involving prescription benzodiazepines stabilized after 2010, they began increasing again in 2019 [9,17]. Several factors could be contributing to increases in benzodiazepine-positive fatal overdoses, including increased fentanyl co-use and changes to substance use, mental health, and the drug market during the COVID-19 pandemic [9,18–21].

The rise in benzodiazepine-positive overdoses poses a public health challenge, especially within the context of the opioid epidemic and the changing drug landscape following the COVID-19 pandemic. In Tennessee, benzodiazepine-positive overdoses increased 46.6% between 2019 and 2020 and remained steady in 2021 [22]. An influx of illicit benzodiazepines in the drug market has been noticed by the State Health Department, but analyses have yet to be performed to identify and understand their use. Therefore, the Tennessee Department of Health sought to examine trends in prescription benzodiazepine and illicit benzodiazepine-positive fatal drug overdoses occurring in the state, with a focus on deaths between 2019–2021.

## Materials and methods

### Data sources

The Tennessee Department of Health routinely conducts fatal drug overdose surveillance through the State Unintentional Drug Overdose Reporting System (SUDORS). SUDORS captures the details associated with fatal overdoses using death certificates, death scene investigations, autopsies, toxicology reports, and Prescription Drug Monitoring Program (PDMP) data, known as the Controlled Substance Monitoring Program Database (CSMD) in Tennessee. SUDORS is nested within the National Violent Death Reporting System (NVDRS) and is funded by an Overdose Data to Action (OD2A) grant from the Centers for Disease Control and Prevention (CDC).

Fatal drug overdoses were selected using the International Classification of Diseases, Tenth Revision (ICD-10 CM) underlying and multiple cause of death codes X40–44, Y10–14, and T36–50 to identify unintentional and undetermined overdose deaths occurring in Tennessee, regardless of the state of residence, from the Tennessee death statistical file. Text search for cause of death fields looking for overdose terms such as “overdose,” “intoxication,” “toxicity,” etc. and drug terms such as “fentanyl,” “heroin, etc. were also used to identify cases when codes were not available on the death certificate. All cases were manually reviewed to confirm their inclusion.

SUDORS cases were linked to their prescription history in CSMD using a unique person identifier created from the name and date of birth. The abstracted data included demographic characteristics, injury and death details, personal circumstances, drug history, death scene investigation, response to drug overdose, prescription fill history for controlled substances, and toxicological data. The cases were abstracted into the Research Electronic Data Capture (REDCap) system by one team member and reviewed by another [23,24]. Prior to submitting the final data to SUDORS, the data underwent a case validation and error report process. Additionally, the cases underwent quality control checks with the CDC [25,26].

Tennessee does not have SUDORS data for all drug overdose decedents prior to 2019. To determine any trends in benzodiazepine-positive overdoses prior to 2019, death certificate data were used for benzodiazepine-positive overdoses from 2012–2021. Prescription benzodiazepine-positive overdoses were defined using the ICD-10 CM underlying cause of death codes X40–44, Y10–14, and multiple cause of death code T42.4 from the Tennessee statistical death file. Illicit benzodiazepine-positive overdoses were defined using a text search of the cause-of-death fields to identify illicit benzodiazepines (Supplemental Table A1).

To obtain more complete information for decedents, SUDORS data were linked to the Hospital Discharge Data System (HDDS) to determine whether any ICD 10-CM codes were present for mental health conditions and substance use disorders. This linkage was conducted using a unique person identifier created based on the name and date of birth. The full list of codes is provided in Supplemental Table A2.

### Study dataset

SUDORS data from 1 January 2019, to 31 December 2021, were obtained from the finalized SAS data file from the CDC for Tennessee. Demographic features included age, marital status, race/ethnicity, sex at birth, and rural/urban residence. Race/ethnicity and sex assigned at birth were determined using the death certificate information. Benzodiazepine-positive overdoses were linked to the CSMD and HDDS to obtain information on the decedents’ mental health, nonfatal overdose, prescription, and substance use disorder history. The data used in the analysis were limited to Tennessee residents 18 years and older with toxicology data to ensure that the most comprehensive information was available for the decedents included in the study.

### Study variables

Benzodiazepines were defined using the benzodiazepine drug class from postmortem toxicology. These classes include prescribed benzodiazepines and illicit benzodiazepines. Benzodiazepine-positive deaths were defined as any benzodiazepine being present in toxicology, regardless of the presence of other substances. Prescription benzodiazepine-positive death was defined as a prescription benzodiazepine being present in toxicology, regardless of the presence of other substances. An illicit benzodiazepine-positive death was defined as illicit benzodiazepine present in toxicology, regardless of the presence of other substances. Prescription benzodiazepines and illicit benzodiazepines were differentiated based on their medical use status in the U.S [9]. Prescription benzodiazepines are approved for medical use in the U.S., while illicit benzodiazepines are not. However, the term “prescription” does not imply that decedents had prescriptions for these medications, as they may have been diverted [9]. A complete list of prescriptions and illicit benzodiazepines identified in SUDORS can be found in Supplemental Table A3.

Race was defined using death certificate data and mutually exclusive coding. White race included all decedents who only had White race recorded on the death certificate. Black race included all decedents who only had Black race recorded on the death certificate. Hispanic ethnicity included decedents of any race with Hispanic ethnicity recorded on the death certificate. Marital status was defined using the following SUDORS categories: never married/single, married/domestic partnership, and widowed/divorced/separated, using death certificate data. Education level was defined using the following SUDORS categories: less than high school, high school graduate or GED, and more than high school, using death certificate data. Rural or urban residences were defined using the 2013 National Center for Health Statistics (NCHS) Urban-Rural Classification Scheme codes. Rural counties were defined as non-metropolitan and non-core counties, as described in the 2013 NCHS Urban-Rural Classification Scheme for Counties [27].

When examining prescription history, several timeframes were defined. “Ever” prescribed was defined as any prescription dating back to 2012 as CSMD prescription data is first available then. Other timeframes included a prescription within 30 days of death, a prescription within 180 days of death, and a prescription within a year of death. These timeframes were used to examine benzodiazepine prescriptions only, as well as overlapping benzodiazepine and opioid prescriptions.

HDDS variables included the history of nonfatal overdose, anxiety disorder, mood disorder, and substance use disorder broken down by type of substance use (i.e. opioid, amphetamine, cocaine, cannabis, inhalant, sedative, and alcohol). The timeframe of the decedents’ most recent nonfatal overdose in comparison to the timing of fatal overdose was also reviewed.

### Statistical analysis

Trends in benzodiazepine-positive fatal overdoses were graphed from 2012–2021 using death certificate data, and from 2019–2021 using SUDORS data. Descriptive statistics were calculated for benzodiazepine-positive overdose deaths and illicit and prescription benzodiazepine-positive deaths using SUDORS data. Crude rates and rate differences were calculated per 100,000 for Tennessee residents for race/ethnicity using 2019–2021 population estimates from the CDC WONDER Single-Race Population Estimates [28]. Chi-square tests of association were conducted where appropriate for differences between prescription and illicit benzodiazepine categories. All analyses were conducted using SAS version 9.4 (SAS Institute, Cary, NC, USA). Statistical significance was set at *p* < 0.05. This study was exempted by the institutional review board of the Tennessee Department of Health.

## Results

**Figure 1.**
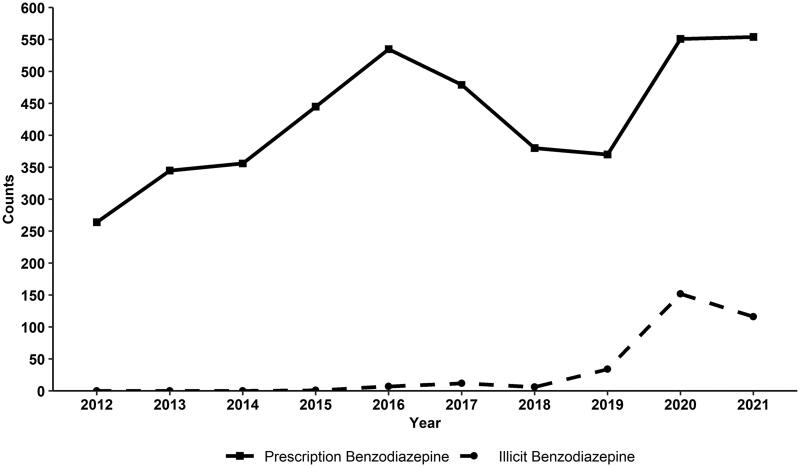
Number of Unintentional and Undetermined benzodiazepine-positive fatal Drug overdoses in Tennessee, 2012–2021. Data is from the Tennessee death Statistical File. Counts of overdose deaths in the Tennessee death Statistical File are slightly different from State Unintentional Drug Overdose Reporting System counts due to differing case definitions. The prescription benzodiazepine category includes deaths coded as T42.4. The illicit benzodiazepine category includes deaths with substances listed in Table A1.

When examining death certificate data from 2012 to 2021, there was an increase in both prescription and illicit fatal benzodiazepine-positive overdoses. Fatal prescription benzodiazepine overdoses peaked in 2016 and then decreased until 2019. Illicit benzodiazepines were first identified in fatal overdoses in Tennessee in 2015, and their identification increased rapidly from 2018 to 2020. The sharpest increase in all benzodiazepine-positive fatal overdoses occurred between 2019 and 2020 (Figure 1).

**Figure 2.**
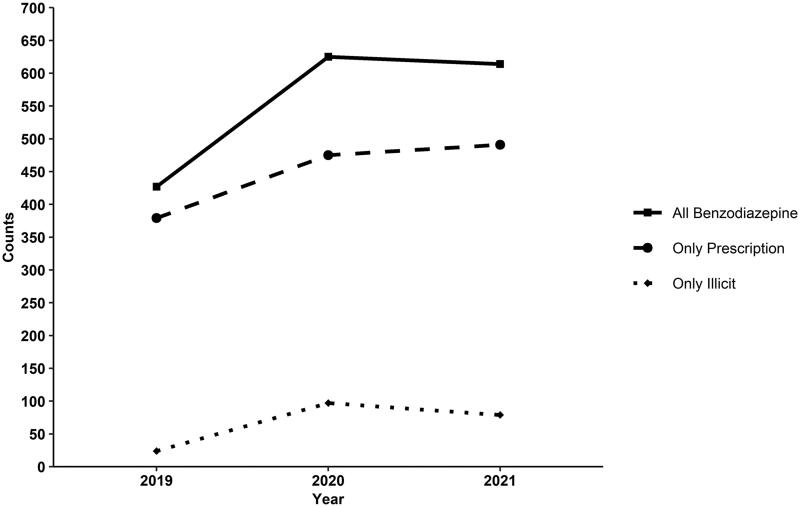
Number of Unintentional and Undetermined benzodiazepine-positive fatal Drug overdoses in Tennessee, 2019–2021. Data is from State Unintentional Drug Overdose Reporting System. Counts of overdose deaths in the Tennessee death Statistical File are slightly different from State Unintentional Drug Overdose Reporting System counts due to differing case definitions.

Using SUDORS data, 1666 benzodiazepine-positive fatal overdoses were identified from 1 January 2019, to 31 December 2021. Fatal prescription benzodiazepine-positive overdoses have increased from 2019 to 2021. Illicit benzodiazepine overdoses increased from 2019 to 2020 and then experienced a slight decrease in 2021 (Figure 2).

### Demographic characteristics of benzodiazepine-positive decedents (Table 1)

Table 1 shows the overall benzodiazepine-positive overdoses, as well as prescription and illicit benzodiazepine-positive overdoses. Decedents with both prescription and illicit benzodiazepines in toxicology were not included in the prescription or illicit benzodiazepine-positive categories but were included in the overall benzodiazepine-positive overdose count. There were 74 decedents with prescription and illicit benzodiazepines on toxicology. Most benzodiazepine-positive decedents were male (58.4%) and lived in an urban area (79.7%). A higher proportion of illicit benzodiazepine-positive decedents were male (66.5% vs. 56.6%) and younger (57.5% vs. 30.9% aged 18–34 years), lived in an urban area (94.0% vs. 76.6%), and had never been married (68.0% vs. 42.7%) compared to prescription benzodiazepine-positive decedents. More prescription benzodiazepine-positive decedents had more than a high school education (30.2% vs. 21.5%) compared to illicit benzodiazepine-positive decedents.

**Table 1.** Demographic characteristics of benzodiazepine-positive decedents, Tennessee residents age 18 and older, TN SUDORS^a^2019–2021, *n* = 1666.

	All Benzodiazepines^b^ N = 1666	Prescription Benzodiazepine (no illicit) N = 1345	Illicit Benzodiazepine (no prescription) N = 200	Chi-square (*p*-value)
Age				
18–24	132 (7.9)	71 (5.3)	48 (24.0)	<0.0001
25–34	449 (27.0)	344 (25.6)	67 (33.5)	
35–44	505 (30.3)	407 (30.3)	55 (27.5)	
45–54	356 (21.4)	318 (23.6)	20 (10.0)	
55^+^	224 (13.5)	205 (15.2)	10 (5.0)	
Sex Assigned at Birth				
Female	693 (41.6)	584 (43.4)	67 (33.5)	0.0080
Male	973 (58.4)	761 (56.6)	133 (66.5)	
Education Level				
Less than High School	352 (21.1)	283 (21.0)	44 (22.0)	0.0330
High School Graduate or GED	806 (48.4)	634 (47.1)	110 (55.0)	
More than High School	482 (28.9)	406 (30.2)	43 (21.5)	
Marital Status				
Married/Domestic Partnership	348 (20.9)	292 (21.7)	31 (15.5)	<.0001
Never Married/Single	777 (46.6)	574 (42.7)	136 (68.0)	
Divorced/Separated/Widowed	519 (31.2)	461 (34.3)	30 (15.0)	
Rural/Urban				
Rural	327 (19.6)	304 (22.6)	11 (5.5)	<.0001
Urban	1327 (79.7)	1030 (76.6)	188 (94.0)	

^a^ Tennessee state unintentional drug overdose reporting system.

^b^ All benzodiazepine category includes benzodiazepines that were generically labeled ‘benzodiazepine’ on toxicology report.

### Mortality rates for benzodiazepine-positive decedents by race (Table 2)

Table 2 shows the overdose mortality rates by race among the benzodiazepine-positive decedents. For all benzodiazepine-positive decedents, the overdose mortality rate was highest among White race, followed by Black race and Hispanic ethnicity. The overdose mortality rate was significantly higher in prescription benzodiazepine-positive decedents compared to illicit benzodiazepine-positive decedents in all demographic categories except for Hispanic ethnicity. The mortality rate for illicit benzodiazepine overdose was slightly higher for Black race decedents when comparing to White race and Hispanic ethnicity (1.10 vs 0.99 and 0.74, per 100,000 Tennessee residents respectively).

**Table 2.** Comparison of overdose rate for benzodiazepine-positive decedents by race, TN SUDORS 2019–2021^a^.

	All Benzodiazepines N = 1666	Prescription Benzodiazepine (no illicit) N = 1345	Illicit Benzodiazepine (no prescription) N = 200	Rate Difference (SE)	*p*-value for rate difference
Race/Ethnicity					
White, non-Hispanic	9.34	7.77	0.99	6.78 (0.24)	<0.0001
Black, non-Hispanic	6.03	4.12	1.10	3.02 (0.39)	<0.0001
Hispanic	1.88	1.06	0.74	0.33 (0.38)	0.3938

^a^Tennessee state unintentional drug overdose reporting system.

Rates were calculated using CDC WONDER’S single-race population estimates from 2019–2021 and are presented per 100,000 Tennessee residents.

Rate difference (SE) was calculated as prescription benzodiazepine death rate minus illicit benzodiazepine death rate.

### Mental health conditions and substance use disorders for benzodiazepine-positive decedents (Table 3)

**Table 3.** Mental health conditions, substance use disorder history, and prior nonfatal overdose for benzodiazepine-positive overdose decedents through linkage with HDDS^c^ data, *N* = 1572.

	All Benzodiazepines N = 1572	Prescription Benzodiazepine (no illicit) N = 1271	Illicit Benzodiazepine (no prescription) N = 187	Chi-square (*p*-value)
Mental Health Conditions				
Anxiety Disorder	715 (45.5)	615 (48.4)	59 (31.6)	<0.0001
Mood Disorder	642 (40.9)	546 (43.0)	61 (32.6)	0.0072
Substance Use Disorder				
Any^a^	837 (53.2)	691 (54.4)	92 (49.2)	0.1888
Alcohol	324 (20.6)	275 (21.7)	35 (18.7)	0.3596
Opioid	504 (32.1)	422 (33.2)	50 (26.7)	0.0801
Amphetamine	237 (15.1)	207 (16.3)	19 (10.2)	0.0323
Cocaine	167 (10.6)	138 (10.9)	17 (9.1)	0.4623
Cannabis	257 (16.4)	207 (16.3)	30 (16.0)	0.9293
Sedative	224 (14.3)	185 (14.6)	27 (14.4)	0.9629
Unspecified	475 (30.2)	393 (30.9)	51 (27.3)	0.3084
Prior Overdose^b^	322 (20.5)	269 (21.2)	35 (18.7)	0.4826
Most recent nonfatal overdose				
1 month	47 (3.0)	38 (3.0)	5 (2.7)	0.8294
3 months	83 (5.3)	65 (5.1)	12 (6.4)	0.4358
6 months	113 (7.8)	92 (7.2)	13 (7.0)	0.9173
1 year	150 (9.5)	121 (9.5)	20 (10.6)	0.5791

^a^ Any does not include alcohol.

^b^ Prior nonfatal overdose dating back to 2012.

^c^Hospital Discharge Data System.

94% of benzodiazepine-positive decedents were linked to the HDDS data. Anxiety was a common diagnosis among benzodiazepine-positive decedents, with 45.5% of decedents having any anxiety disorder. Around half of all decedents (53.2%) had any substance use disorder, and 32.1% had an opioid use disorder. Compared with prescription benzodiazepine-positive decedents, illicit benzodiazepine-positive decedents had lower percentages of any anxiety and any mood disorder diagnosis (31.6% vs. 48.4% and 32.6% vs. 43.0%, respectively). There was no significant difference in any specific type of substance use disorder except amphetamine use disorder, which was more prevalent among prescription benzodiazepine-positive decedents (16.3% vs. 10.2%). Overall, 20.5% of benzodiazepine-positive fatal overdose decedents had a prior nonfatal hospital-treated overdose dating back to 2012. When looking at prior overdose at 1 month, 3 months, 6 months, and 1 year, and dating back to 2012, there was no significant difference by benzodiazepine type. Within 1 month of death, 3% of all benzodiazepine-positive fatal overdoses decedents experienced a nonfatal overdose treated in a hospital setting.

### Toxicology information for benzodiazepine-positive decedents (Table 4)

**Table 4.** Toxicology by class and individual substance for benzodiazepine-positive decedents, TN SUDORS^a^ 2019–2021, *N* = 1666.

	All Benzodiazepines N = 1666	Prescription Benzodiazepine (no illicit) N = 1345	Illicit Benzodiazepine (no prescription) N = 200	Chi-square (*p*-value)
Drug classes^b^				
Opioids	1554 (93.3)	1246 (92.6)	192 (96.0)	0.0807
Stimulants	833 (50.0)	663 (49.3)	97 (48.5)	0.8341
Antidepressants	453 (27.2)	407 (30.3)	27 (13.5)	<0.0001
Alcohol	332 (19.9)	277 (20.6)	37 (18.5)	0.4922
Cannabis	539 (32.4)	393 (29.2)	96 (48.0)	<0.0001
Individual Substances‡				
Fentanyl	1187 (71.3)	923 (68.6)	165 (82.5)	<.0001
Rx Opioid	79 (4.7)	70 (5.2)	7 (3.5)	0.3014
Methamphetamine	542 (32.5)	449 (33.4)	54 (27.0)	0.0723
Cocaine	216 (13.0)	157 (11.7)	33 (16.5)	0.0524
Heroin	251 (15.1)	196 (14.6)	21 (10.5)	0.1210

^a^ Tennessee state unintentional drug overdose reporting system.

^b^ Substances are not mutually exclusive.

Almost all benzodiazepine-positive overdoses also involved opioids (93.3%) and often involved fentanyl (71.3%). Prescription benzodiazepine-positive overdoses more often involved antidepressants than illicit benzodiazepine-positive overdoses (30.3% vs. 13.5%) and less often involved cannabis (29.2% vs. 48.0%). Fentanyl was the only substance for which there was a significant difference between the groups, with more illicit benzodiazepine-positive overdoses also involving fentanyl (82.5% vs. 68.6%).

### Prescription information for benzodiazepine-positive decedents (Table 5)

**Table 5.** Prescription information for benzodiazepine-positive deaths through linkage with CSMD^a^ data, *N* = 1517.

	All Benzodiazepines N = 1517	Prescription Benzodiazepine (no illicit) N = 1243	Illicit Benzodiazepine (no prescription) N = 165	Chi-square *p*-value
Benzodiazepine prescription				
Ever^b^	671 (44.3)	596 (48.0)	40 (24.2)	<0.0001
Within 365 days of death	435 (28.7)	400 (32.2)	13 (7.9)	<0.0001
Within 180 days of death	391 (25.8)	363 (29.2)	9 (5.5)	<0.0001
Within 30 days of death	288 (19.0)	270 (21.7)	3 (1.8)	<0.0001
Overlapping Opioid and Benzodiazepine Prescription				
Ever^b^	554 (36.5)	496 (39.9)	31 (18.8)	<0.0001
Within 365 days of death	283 (18.7)	266 (21.4)	5 (3.0)	<0.0001
Within 180 days of death	228 (15.0)	215 (17.3)	5 (3.0)	<0.0001
Within 30 days of death	145 (9.6)	136 (11.0)	2 (1.2)	<0.0001

^a^ Controlled substances monitoring program data.

^b^ Ever dates back to 2012.

All benzodiazepine-positive decedents had high proportions of ever having a benzodiazepine prescription (44.3%) or ever having an overlapping benzodiazepine and opioid prescription (36.5%). However, all prescription categories showed significant differences between prescription and illicit benzodiazepine-positive decedents. Prescription benzodiazepine-positive decedents more often had a benzodiazepine prescription as well as an overlapping benzodiazepine and opioid prescription at all given time points prior to death. Additionally, when linking benzodiazepine-positive overdoses to the HDDS and CSMD data, co-occurring mental health conditions and prescription medications differed by benzodiazepine type (Supplemental Table A4).

## Discussion

This study explored the characteristics of benzodiazepine-positive drug overdose decedents in Tennessee through the linkage of SUDORS fatal overdose, HDDS, and CSMD data. In recent years, the number of benzodiazepine-positive fatal overdoses has increased. Between 2019 and 2021, illicit benzodiazepine-positive fatal overdoses increased by 225%, which is more than the 34% increase in prescription benzodiazepine-positive fatal overdoses during the same period. This aligns with recent literature suggesting that the use of novel psychoactive substances is increasing worldwide [29]. However, illicit benzodiazepine-positive fatal overdoses have decreased by 18% from 2020 to 2021, while prescription benzodiazepine-positive fatal overdoses have increased slightly (Figure 2). This aligns with reports of increased illicit benzodiazepine use during the height of the COVID-19 pandemic [30]. The continued increase in prescription benzodiazepines, coupled with the emergence of illicit benzodiazepines, suggests a need for further investigation into benzodiazepine overdose prevention efforts.

Increased co-use of benzodiazepines with illicit fentanyl is likely a prominent cause of benzodiazepine-positive overdose. Postmortem toxicology in Tennessee showed that fentanyl was present in 69% of prescription benzodiazepine-positive overdoses and 83% of illicit benzodiazepine-positive overdoses. This corresponds with research showing that fatal benzodiazepine-positive overdoses typically involve fentanyl as well [9]. Involvement of fentanyl in overdose deaths has increased exponentially since 2013, resulting in a new wave of the overdose epidemic[1]. Reports of illicit benzodiazepines also began increasing around this time, with etizolam first being reported to the Drug Enforcement Agency’s (DEA) National Forensic Laboratory Information System (NFLIS) in 2012 [12]. While prescription benzodiazepine overdoses have not increased as rapidly as illicit benzodiazepine overdoses, they have continued to increase across the country as the polydrug overdose crisis continues [9]. While benzodiazepines rarely result in fatal overdoses when used alone, concurrent use with other central nervous system (CNS) depressants such as fentanyl can cause depressed breathing, impaired cognitive function, slowed heart rate, sedation, and death [31,32].

In addition to fentanyl, an increase in benzodiazepine-positive overdoses could be related to the COVID-19 pandemic. Studies have shown that COVID-19 increased mental health challenges, especially among people with substance use disorder [33,34]. It is possible that social isolation, anxiety, and reduced access to drug treatment facilities resulted in increased substance use [18]. Supply chain disruptions could have contributed to the rise in diversions of prescription benzodiazepines [35]. Our data demonstrates a sharp increase in benzodiazepine-positive overdoses during 2020, corresponding with research that more people may have sought out benzodiazepines to treat pandemic-related anxiety [35]. This increase also corresponds to recent reports of illicit benzodiazepines being found as adulterants in the unregulated opioid supply in North America [15]. Drug seizures involving illicit benzodiazepines increased by 157% from 2019 to 2020, indicating the increased presence of these substances on the illicit drug market [30]. Most people who use substances are unaware of the presence of illicit benzodiazepines in the opioid drug supply, thus increasing the risk of fatal overdose [36].

In terms of postmortem toxicology, in addition to fentanyl, antidepressants, and cannabis showed statistically significant differences between prescription and illicit benzodiazepine-positive decedents. Cannabis was more commonly found with illicit benzodiazepines, consistent with reports that marijuana and benzodiazepine use increased during the pandemic to cope with stress [37]. An additional study also supported our observation of low antidepressant co-use among illicit benzodiazepine-positive overdose decedents [9]. This corresponds with circumstance variables showing that illicit benzodiazepine-positive decedents had significantly lower proportions of anxiety and mood disorders compared to prescription benzodiazepine-positive decedents. While illicit benzodiazepine-positive decedents also had fewer prescriptions overall, only 22% of prescription benzodiazepine-positive decedents had a benzodiazepine prescription in the 30 days prior to death, suggesting mostly non-medical use of prescription benzodiazepines. This is in accordance with evidence that benzodiazepines are one of the most commonly misused prescription medications and that non-medical use of benzodiazepines increased during the pandemic [38,39]. Interestingly, there was no difference in the proportion of decedents with prescription opioids or substance use disorders by benzodiazepine type. This contrasts with evidence showing more prescription opioids among prescription benzodiazepine-positive overdose decedents compared to illicit benzodiazepine-positive decedents[9]. This may further support the theory that prescription benzodiazepines are being used non-medically.

When looking at the race of decedents, we found that White race had the highest overdose mortality rate among prescription benzodiazepine-positive decedents, while Black race had the highest overdose mortality rate among illicit benzodiazepine-positive decedents, supporting research showing racial differences in illicit versus prescription drug use [40,41]. Previous studies have also demonstrated that benzodiazepines are more commonly prescribed and used among White people compared to all other races [2,40,42,43]. While illicit drugs now account for most fatal overdoses among all races, they disproportionately impact ethnic minorities [44]. In Tennessee, rates of fentanyl-positive fatal overdose were still higher among White race compared to Black race in 2019 but shifted starting in 2020 [22]. This recent increase among the Black population demonstrates the need for culturally appropriate prevention and treatment methods for benzodiazepine-positive overdose [45].

There are several prevention efforts that could be implemented to curb benzodiazepine-positive fatal overdoses. In our sample, nearly half of all prescription benzodiazepine-positive decedents had any anxiety disorder, suggesting that these decedents interacted with the mental health system prior to death. Health care providers should continue to look for signs of benzodiazepine dependence and educate patients on appropriate benzodiazepine use [3]. Previous research has shown that educating patients on the risks of benzodiazepine use can reduce misuse and encourage benzodiazepine discontinuation [46]. Since 20% of all benzodiazepine-positive fatal overdoses had a prior nonfatal overdose treated in a hospital setting, linkage to care efforts from the emergency department are critical. Rapid toxicology testing at medical facilities could aide providers in identifying a benzodiazepine-involved overdose. For illicit benzodiazepines, it is important to increase the capacity of regional forensic centers to test for emerging substances. Laboratories in Tennessee should investigate non-targeted testing methods such as data mining and sample mining to better identify novel psychoactive substances [47,48].

Harm reduction strategies can also be implemented to prevent overdoses involving benzodiazepines. The initiation of drug checking services, where substances can be tested to determine their actual composition, in Tennessee could provide an opportunity to better understand the current state of the illicit drug supply. Drug checking programs have effectively identified NPS in parts of Europe and North America [49–51]. Although fentanyl test strips (FTS) were decriminalized in Tennessee in 2022, there are no other drug-checking services in the state [52]. Additional drug checking could be initiated at syringe service programs (SSPs) in Tennessee, where people who use substances could be connected with other services such as HIV testing and substance use disorder treatment [53,54].

Additionally, naloxone distribution and training continue to be essential strategies for preventing overdose-related deaths. Given the high proportion of fentanyl co-involvement in benzodiazepine-positive overdoses, naloxone is likely to reduce mortality in this population. However, naloxone does not reverse the effects of benzodiazepines. Education is needed on the dangers of combining opioids and benzodiazepines, and training programs should focus on identifying the signs of an opioid overdose versus a benzodiazepine overdose so that a patient can be treated accordingly [9].

### Limitations

Our study has several limitations. Tennessee’s medicolegal death investigation is organized in a decentralized system consisting of 95 county medical examiners and 5 regional forensic centers, each with its own policies and investigative authority regarding autopsies. As such, the state public health network provides training, support, and recommendations but has no authority over individual centers, causing the level of detail for scene investigation to vary between reports. Additionally, because illicit benzodiazepines are not included in the generic toxicology panel, they were likely underestimated in this analysis. An effort was made to acquire the most comprehensive data for each decedent by linking HDDS and CSMD data; however, our data are limited to what has been reported to the medical community. Any conditions for which decedents did not seek medical care were most likely not captured. Another limitation was that the COVID-19 pandemic was at its height during the study period. Some of these data are most likely indicative of the pandemic and may not represent a progressive trend over time. Data on socioeconomic status is limited to decedent education level. Lastly, the data are specific to the state of Tennessee, and the results are not representative of national trends.

## Conclusions

Trends in benzodiazepine overdoses have continued to evolve in Tennessee. This study identified the populations most at risk for benzodiazepine overdose. Nearly half of the decedents had a documented mental health condition in hospital discharge records, confirming the importance of adequate access to mental health care. Additionally, 3.0% of decedents had nonfatal overdose treated in the hospital setting within the month before death, confirming the importance of linkage to care efforts in the emergency department. This analysis can inform local and regional public health workers to implement focused prevention and intervention efforts such as the risks of using multiple substances or obtaining prescription benzodiazepines illicitly. Additionally, emphasis should be placed on the importance of comprehensive mental health care once diagnosed with a mental health condition, of course this relies on the availability of such care to patients. The differences between prescription and illicit benzodiazepine-positive fatal overdoses indicate the need to develop substance-specific prevention and treatment strategies.

## Supplementary Material

Supplemental Material

## Data Availability

The data supporting these findings are available upon request from the Tennessee Department of Health upon publication.
