# Supplementary material for: Exploring trends in benzodiazepine-positive fatal drug overdoses in Tennessee, 2019–2021
Source: Ann Med. 2023 Dec 1;55(2):2287194. doi: 10.1080/07853890.2023.2287194 (PMC10836290; doi:10.1080/07853890.2023.2287194)
Supplement: Supplemental Material [file IANN_A_2287194_SM6593.docx]

**Appendices**

| Table A1. Illicit benzodiazepines searched for in Tennessee Death Statistical File |
| --- |
| Bromazolam |
| Clonazolam |
| Delorazepam |
| Diclazepam |
| Desalkylgidazepam |
| Deschloroalprazolam |
| Etizolam |
| Flualprazolam |
| Flubromazepam |
| Flubromazolam |
| Phenazolam |
| Clobazam |

| Table A2. ICD-10 codes used for mental health condition and substance use disorder in HDDS^§^ linkage | |
| --- | --- |
| ICD-10 Code | Description |
| F11-F16, F18-F19 | Any Substance Use Disorder, not alcohol |
| F10 | Alcohol Use Disorder |
| F11 | Opioid Use Disorder |
| F12 | Cannabis Use Disorder |
| F13 | Sedatives Use Disorder |
| F14 | Cocaine Use Disorder |
| F15 | Amphetamine Use Disorder |
| F16 | Hallucinogen Use Disorder |
| F18 | Inhalant Use Disorder |
| F19 | Unspecified Use Disorder |
| F30-F39 | Manic episode, bipolar disorder, depressive episode, major depressive disorder, recurrent, persistent mood affective disorders, unspecified mood affective disorders |
| F40-F49 | Phobic anxiety disorders, panic anxiety disorders, adjustment disorders, post-traumatic stress disorder, reaction to severe stress, obsessive compulsive disorder, dissociative and conversion disorders, somatoform disorders, general anxiety disorder |
| ^§^Hospital Discharge Data System | |

| Table A3. Frequency and percentage of all SUDORS prescription benzodiazepine substances and illicit benzodiazepine substances^*^ | | | |
| --- | --- | --- | --- |
|  | 2019  N=427 | 2020  N=625 | 2021  N=614 |
|  | N (%) | N (%) | N (%) |
| Prescription Benzodiazepine |  |  |  |
| Alprazolam | 218 (51.1) | 270 (43.2) | 261 (42.5) |
| Chlordiazepoxide | 10 (2.3) | 10 (1.6) | 6 (1.0) |
| Clonazepam | 118 (27.6) | 137 (21.9) | 159 (25.9) |
| Diazepam | 64 (15.0) | 94 (15.0) | 89 (14.5) |
| Nordiazepam | 83 (19.4) | 126 (20.2) | 112 (18.2) |
| Lorazepam | 15 (3.5) | 27 (4.3) | 28 (4.6) |
| Midazolam | 7 (1.6) | 6 (1.0) | 15 (2.4) |
| Oxazepam | 14 (3.3) | 17 (2.7) | 10 (1.6) |
| Temazepam | 12 (2.8) | 16 (2.6) | 19 (3.1) |
| Illicit Benzodiazepine |  |  |  |
| Bromazolam | 0 (0.0) | 0 (0.0) | 8 (1.3) |
| Clonazolam | 0 (0.0) | 2 (0.3) | 7 (1.1) |
| Etizolam | 24 (5.6) | 82 (13.1) | 84 (13.7) |
| Flualprazolam | 7 (1.6) | 41 (6.6) | 5 (0.8) |
| Flubromazolam | 2 (0.5) | 14 (2.2) | 7 (1.1) |
| ^*^Substances are not mutually exclusive and do not necessarily reflect the true substance ingested due to drug metabolism. | | | |

| Table A4. Comparison of types of benzodiazepine-positive overdose with types of mental health condition and prescription medication, linkage of HDDS^§^ and CSMD^*^ | | | | |
| --- | --- | --- | --- | --- |
|  | All Benzodiazepines  N=1,468 | Prescription Benzodiazepine (no illicit)  N=1,203 | Illicit Benzodiazepine (no prescription)  N=159 | Chi-square p-value |
| Anxiety disorder and benzodiazepine prescription | 395(26.9) | 51(29.2) | 21(13.2) | <0.0001 |
| Mood disorder and  benzodiazepine prescription | 350(23.8) | 316(26.3) | 18(11.3) | <0.0001 |
| Anxiety disorder and overlapping benzodiazepine and opioid prescription | 347(23.7) | 309(25.7) | 19(12.0) | <0.0001 |
| Mood disorder and overlapping benzodiazepine and opioid prescription | 309(21.1) | 280(23.3) | 16(10.1) | <0.0001 |
| ^§^Hospital Discharge Data System  ^*^Controlled Substance Monitoring Database | | | | |
